# In vivo pharmacokinetic/Pharmacodynamic modeling of Enrofloxacin against *Escherichia coli* in broiler chickens

**DOI:** 10.1186/s12917-018-1698-3

**Published:** 2018-11-29

**Authors:** Xia Xiao, Lijie Jiang, Weixuan Lan, Yongjia Jiang, Zhiqiang Wang

**Affiliations:** 1 College of Veterinary Medicine, Yangzhou University, Yangzhou, 225009 Jiangsu People’s Republic of China; 2 Jiangsu Co-innovation Center for Prevention and Control of Important Animal Infectious Diseases and Zoonoses, Yangzhou, Jiangsu 225009 People’s Republic of China; 3 Institutes of Agricultural Science and Technology Development, 48 East Wenhui Road, Yangzhou, Jiangsu 225009 People’s Republic of China

**Keywords:** *Escherichia coli*, Enrofloxacin, In vivo, PK/PD modeling, Chicken

## Abstract

**Background:**

Systemic *Escherichia coli* infections cause early mortality of commercial broiler chickens. Although enrofloxacin has long been used in poultry, the in vivo pharmacokinetic/pharmacodynamic (PK/PD) relationship of enrofloxacin against *E. coli* is unclear. The present study aimed to establish an in vivo PK/PD model of enrofloxacin against *E. coli* in seven-day-old chicks and to ascertain whether the selection of target organ for PD determination is critical for parameter magnitude calculation in enrofloxacin PK/PD modeling.

**Results:**

The in vivo effectiveness of enrofloxacin against *E. coli* in different organs varied, with the *E*_*max*_ ranging from − 4.4 to − 5.8 Log_10_ colony forming units (cfu)/mL or cfu/g. Both the surrogate AUC_0–24_/MIC of enrofloxacin or AUC_0–24_/MIC of the combination of enrofloxacin and ciprofloxacin correlated well with effectiveness in each organ. The AUC_0–24_/MIC ratio of the combination of enrofloxacin and ciprofloxacin producing bactericidal and elimination effects were 21.29 and 32.13 in blood; 41.68, and 58.52 in the liver; and 27.65 and 46.22 in the lung, respectively.

**Conclusions:**

The in vivo effectiveness of enrofloxacin against *E. coli* in different organs was not identical after administration of the same dosage. To describe the magnitude of PK/PD parameter exactly, bacterial loading reduction in different organs as PD endpoints should be evaluated and compared in PK/PD modeling. The selection of a target organ to evaluate PDs is critical for rational dosage recommendation.

## Background

It is estimated that 50% of total poultry loses could be attributed to first week mortalities [1]. Among them, over 50% of mortalities are caused by bacterial infections, primarily *Escherichia coli* [2]. Systemic *E. coli* infections contribute significantly to the early mortality of commercial broiler chickens [3]. However, because of the high diversity in virulence-associated genes and serotypes, effective vaccines against *E. coli* challenge are rare [4]. Until now, using antimicrobials has been the main strategy to control *E. coli* infections in the poultry industry.

Enrofloxacin, a second-generation fluoroquinolone, is commonly used in chickens because of its favorable pharmacokinetic (PK) profile and its excellent activity against gram-negative aerobic bacteria and some gram-positive bacteria [5, 6]. However, with the extensive use of enrofloxacin, resistance has emerged [7]. Enrofloxacin is metabolized to ciprofloxacin, which is used clinically in humans, and there are reports showing that resistance genes for fluoroquinolones could transfer to other organisms under antimicrobial pressure [8, 9]. Thus, the non-rational usage of enrofloxacin runs the risk of leading to bacterial resistance and potential health hazards in humans [10–12]. Thus, there is a growing need to optimize the use of enrofloxacin.

Optimizing the use of an antimicrobial should be based on a good understanding of its PK and pharmacodynamic (PD) relationship in target animals against specific bacterial species [13]. Although ex vivo PK/PD modeling of enrofloxacin has been evaluated in buffalo calves, swine, and chickens against *E. coli*, *Pasteurella multocida*, and *Salmonella typhimurium* [14–20], to the best of our knowledge, there are no in vivo PK/PD modeling studies of enrofloxacin against *E. coli* in chicks. In vivo PK/PD modeling has great advantages over ex vivo modeling in describing the PK/PD relationship [21], especially for enrofloxacin, whose metabolite, ciprofloxacin, (another fluoroquinolone) has almost the same potency as enrofloxacin. Therefore, for PK/PD modeling of enrofloxacin, it is important to involve its metabolism to ciprofloxacin in the modeling. According to our previous study, the selection of the target organ for PD determination is critical for parameter magnitude calculation in antimicrobial PK/PD modeling [22]. Whether this is true for enrofloxacin requires further investigation.

In the present study, to further understand the PK/PD relationship of enrofloxacin, especially whether the selection of a target organ for PD determination is critical for parameter magnitude calculation in PK/PD modeling, broilers were used as an animal model. The following aspects were investigated: (1) The pharmacokinetics of enrofloxacin and its metabolism to ciprofloxacin were determined at three different dosage administrations; (2) the in vivo PK/PD modeling of enrofloxacin against *E. coli* was developed using the reductions in the bacterial burden in the blood, liver, and lung as the PD endpoints; (3) whether the concentration of ciprofloxacin influence the in vivo PK/PD modeling of enrofloxacin was evaluated; and (4) the corresponding magnitude of PK/PD parameters for a certain efficacy were determined.

## Results

### In vitro susceptibility

The MICs of enrofloxacin and ciprofloxacin against *E. coli* O78 were the same (0.5 μg/mL). The corresponding MBC values were 0.5 and 1 μg/mL respectively. The MIC and MBC of enrofloxacin in serum were identical (0.5 μg/mL). The MPC of enrofloxacin was 3.2 μg/mL **(**Table 1).

**Table 1:** MIC and MPC values (μg/mL) of enrofloxacin and ciprofloxacin against *E. coli* O78 in Mueller–Hinton broth and serum

Parameters (μg/mL)	ENR (broth)	ENR (serum)	CIP (broth)
MIC	0.5	0.5	0.5
MBC	0.5	0.5	1
MPC	3.2	__	__

*MIC* minimum inhibitory concentration, *MPC* mutant prevention concentration, *MBC* minimum bactericidal concentration, *ENR* enrofloxacin, *CIP* ciprofloxacin

### *E. coli* infection model

Clinical signs of colibacillosis, such as depression, decreased feeding, diarrhea, and fever were observed 24 h after challenge with *E. coli* O78. After dissection, perihepatitis and pericarditis were obvious. The bacteria load in the blood, liver, and lung were 7.2 ± 0.92 Log_10_ cfu/mL, 6.4 ± 0.14 Log_10_ cfu/g, and 6.1 ± 0.17 Log_10_ cfu/g respectively. The bacterial load in the three organs was similar among different chicks. The death rate was 10%.

### Pharmacokinetics

**Fig. 1:**
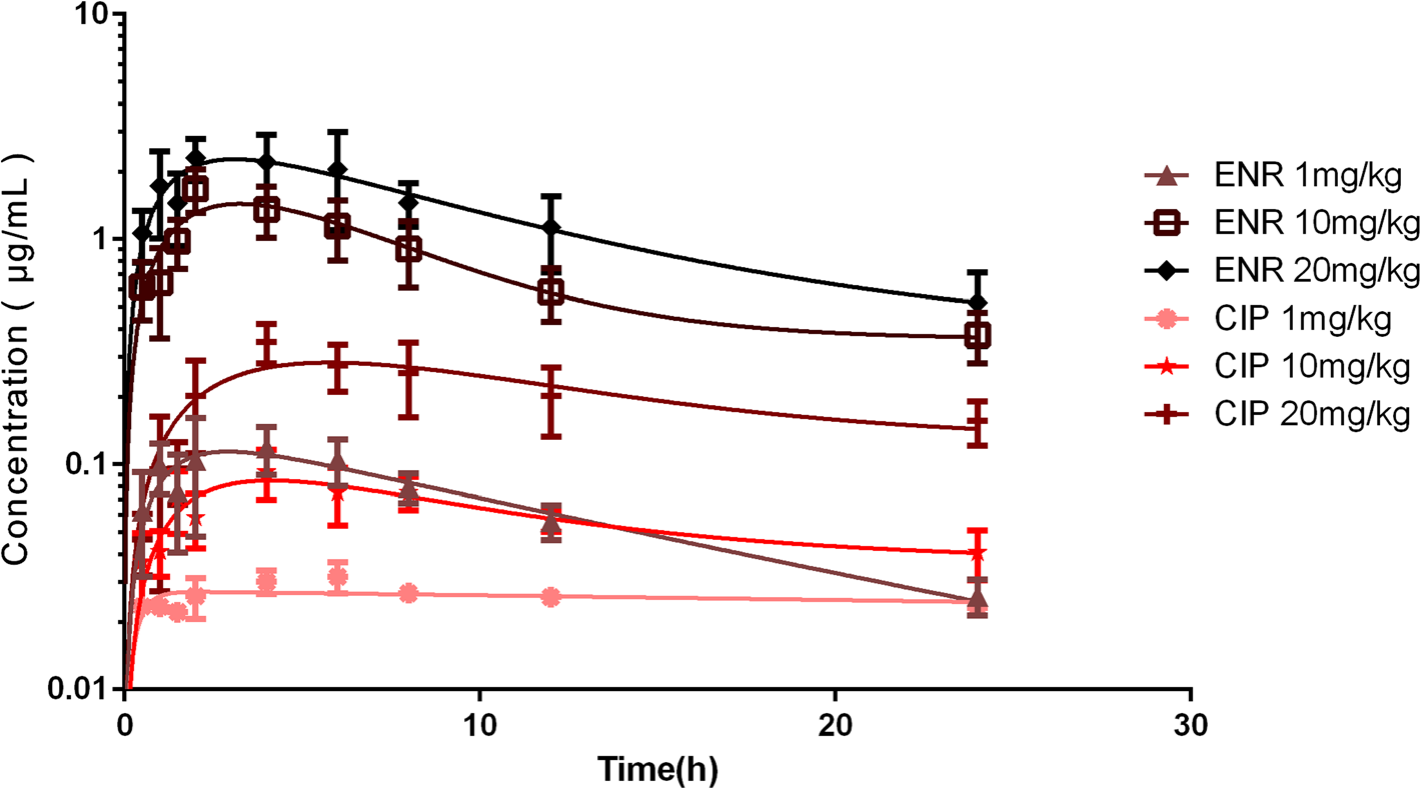
The time-concentration profiles of enrofloxacin or ciprofloxacin in serum after oral administration of enrofloxacin at doses of 1, 10, and 20 mg/kg in *E. coli* O78-infected chicks. [enrofloxacin (ENR); ciprofloxacin (CIP)]

**Fig. 2:**
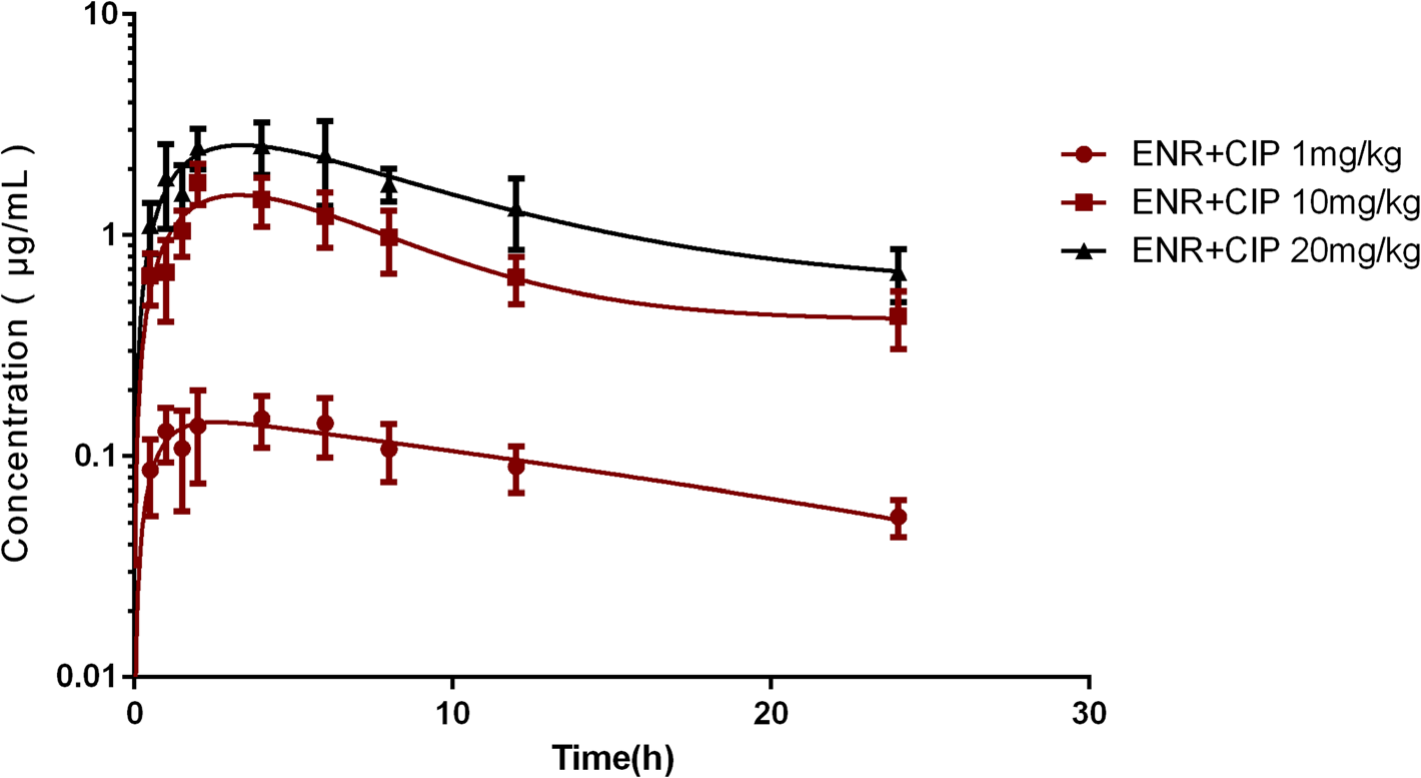
The time-concentration profiles of the combination of enrofloxacin and ciprofloxacin in serum after oral administration of enrofloxacin at doses of 1, 10, and 20 mg/kg in *E. coli* O78-infected chicks. [enrofloxacin (ENR); ciprofloxacin (CIP)]

The serum drug concentration-time profiles of enrofloxacin and ciprofloxacin after enrofloxacin administration at three dosages are illustrated in Fig. 1 and Fig. 2. The PK parameters of enrofloxacin and ciprofloxacin are shown in Table 2 and Table 3. The time of peak concentration (T_max_) for enrofloxacin and ciprofloxacin were about 3.3~ 3.4 h and 4.3~ 5 h, respectively, with peak concentrations (C_max_) of 0.16, 1.76, and 2.86 μg/mL for enrofloxacin at 1, 10, and 20 mg/kg, respectively; and of 0.03, 0.10, and 0.37 μg/mL at corresponding doses for ciprofloxacin. The C_max_ of ciprofloxacin was much lower and emerged later than enrofloxacin. The AUC_0–24_ of enrofloxacin at 1, 10, and 20 mg/kg b.w. was 1.64, 17.95, and 30.07 h, respectively, and the corresponding values for ciprofloxacin were 0.61, 1.42, and 4.93 h, respectively. The AUC_0–24_ values of enrofloxacin were 2.6, 12.6, and 6.09 times higher than those of ciprofloxacin at doses of 1, 10, and 20 mg/kg b.w. respectively. Dose proportionality was observed for the AUC_0–24_ of enrofloxacin and ciprofloxacin in the range of 1–20 mg/kg with r^2^ of 0.9868 and 0.9035, respectively. Thus, the AUC_0–24_ of other doses between 1 and 20 mg/kg could be calculated.

**Table 2:** Pharmacokinetic parameters of enrofloxacin in serum after oral administration of 1, 10, and 20 mg/kg body weight enrofloxacin in *E. coli* O78-infected chicks (mean and SD, *n* = 10)

Parameters	1 mg/kg	10 mg/kg	20 mg/kg
C_max_ (μg/mL)	0.16 ± 0.041	1.76 ± 0.32	2.86 ± 0.52
T_max_ (h)	3.35 ± 1.8	3.4 ± 2.12	3.3 ± 1.77
T_1/2β_ (h)	10.75 ± 1.8	11.44 ± 1.41	9.78 ± 1.26
AUC_0–24_ (h·μg/mL)	1.64 ± 0.28	17.95 ± 2.45	30.07 ± 4.64
CL/F (mL/h/kg)	488 ± 114	420 ± 85	548 ± 114

*C*_*max*_ peak concentration, *T*_*max*_ time of peak concentration, *T*_*1/2β*_ elimination half-life, *AUC*_*0–24*_ area under the concentration-time curve from 0 to 24 h, *CL/F* oral clearance

**Table 3:** Pharmacokinetic parameters of ciprofloxacin in serum after oral administration of 1, 10, and 20 mg/kg body weight enrofloxacin in *E. coli* O78-infected chicks (mean and SD, *n* = 10)

Parameters	1 mg/kg	10 mg/kg	20 mg/kg
C_max_ (μg/mL)	0.03 ± 0.001	0.10 ± 0.03	0.37 ± 0.05
T_max_ (h)	5 ± 1.05	4.3 ± 1.98	4.4 ± 1.58
T_1/2β_ (h)	27.25 ± 7.76	19.51 ± 2.60	18.77 ± 4.11
AUC_0–24_ (h·μg/mL)	0.61 ± 0.03	1.42 ± 0.15	4.93 ± 0.76
CL/F (mL/h/kg)	0.67 ± 0.11	3.96 ± 0.78	2.26 ± 0.46

*C*_*max*_ peak concentration, *T*_*max*_ time of peak concentration, *T*_*1/2β*_ elimination half-life, *AUC*_*0–24*_ area under the concentration-time curve from 0 to 24 h, *CL/F* oral clearance

### In vivo PK/PD analysis

The AUC_0–24_/MIC ratios of enrofloxacin against *E. coli* O78 for doses of 1, 2, 5, 7.5, 10, 12.5, 15, and 20 mg/kg were 5.27, 8.26, 17.20, 24.66, 32.11, 39.57, 47.02, and 61.93 h respectively. The corresponding AUC_0–24_/MIC values of ciprofloxacin were 0.36, 0.82, 2.2, 3.35, 4.50, 5.65, 6.80, and 13.24 h, respectively.

**Fig. 3:**
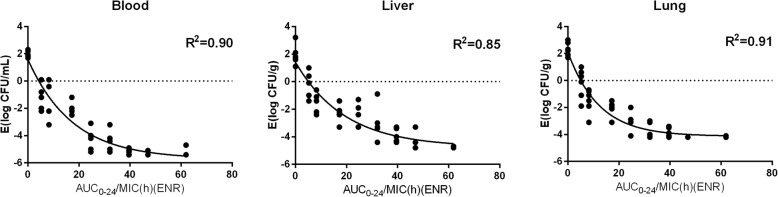
Sigmoid maximum effect (*E*_*max*_) relationship between the in vivo antimicrobial effect against *E. coli* O78 in three tissues of chicken and the area under the concentration-time curve from 0 to 24 h (AUC_0–24_)/minimum inhibitory concentration (MIC) ratio of enrofloxacin. [enrofloxacin (ENR)]

**Fig. 4:**
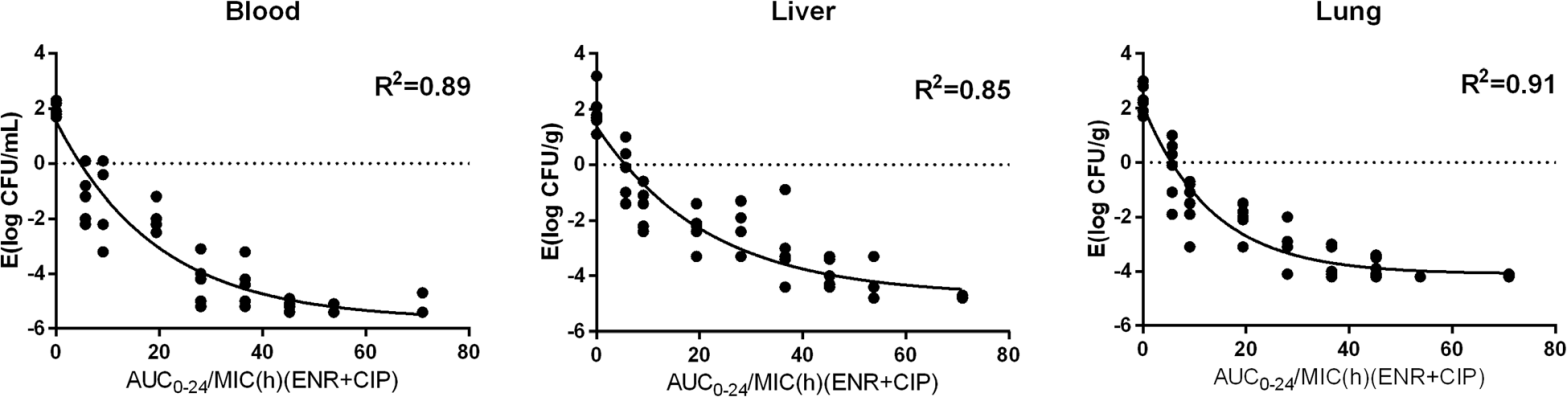
Sigmoid maximum effect (*E*_*max*_) relationship between the in vivo antimicrobial effect against *E. coli* O78 in three tissues of chicken and the area under the concentration-time curve from 0 to 24 h (AUC_0–24_)/minimum inhibitory concentration (MIC) ratio of enrofloxacin and ciprofloxacin. [enrofloxacin (ENR); ciprofloxacin (CIP)]

The relationships between the effectiveness (bacteria loading reduction) of enrofloxacin in different organs and PK/PD indices of enrofloxacin, or the combination of enrofloxacin and ciprofloxacin, are shown in Fig. 3 and Fig. 4. The surrogate AUC_0–24_/MIC correlated well with effectiveness in each organ, with r^2^ values greater than 0.85. The in vivo effectiveness of enrofloxacin against *E. coli* in different organs varied, with *E*_*max*_ ranging from − 4.4 to − 5.8 Log_10_ cfu/mL. Using the PK of enrofloxacin for simulation, then the AUC_0–24_/MIC values of enrofloxacin for the bactericidal effect in the blood, liver, and lung were 19.32, 32.15, and 23.41, respectively. The slopes were 2.58, 2.36, and 3.01 for the blood, liver, and lung, respectively (Table 4). Using the combined PK of enrofloxacin and ciprofloxacin for simulation, then the AUC_0–24_/MIC values for the bactericidal effect in the blood, liver, and lung were 21.29, 41.68, and 27.65, respectively (Table 5). The slopes were 4.19, 2.67, and 4.86 for the blood, liver, and lung, respectively.

**Table 4:** In vivo PK/PD parameters of enrofloxacin in *E. coli* O78-infected chicks using the concentration of enrofloxacin for simulation

Parameters	Blood	Liver	Lung
*E*_*max*_ (Log _10_ cfu/mL)	−5.8	− 4.7	−4.4
EC_50_	16.61	16.93	10.33
E_0_ (Log _10_ cfu/mL)	1.78	1.68	2.29
AUC_0–24_/MIC for 1 log_10_ cfu/mL killing	9.29	12.42	9.1
AUC_0–24_/MIC for 3 log_10_ cfu/mL killing	19.32	32.15	23.41
AUC_0–24_/MIC for 4 log_10_ cfu/mL killing	28.17	52.52	56.36
Slope (N)	2.58	2.36	3.01

*E*_*max*_ maximum effect, *EC*_*50*_ concentration of a drug that gives half-maximal response, *E*_*0*_ concentration at baseline, *AUC*_*0–24*_ area under the concentration-time curve from 0 to 24 h, *cfu* colony forming units

**Table 5:** In vivo PK/PD parameters of enrofloxacin in *E. coli* O78-infected chicks using the combined concentration of enrofloxacin and ciprofloxacin for simulation

Parameters	Blood	Liver	Lung
*E*_*max*_ (Log _10_ cfu/mL)	−5.8	−4.7	−4.4
EC_50_	18.28	17.66	13.59
E_0_ (Log _10_ cfu/mL)	1.78	1.68	2.29
AUC_0–24_/MIC for 1 log_10_ cfu/mL killing	9.74	14.01	9.49
AUC_0–24_/MIC for 3 log_10_ cfu/mL killing	21.29	41.68	27.65
AUC_0–24_/MIC for 4 log_10_ cfu/mL killing	32.13	60.52	57.24
Slope (N)	4.19	2.67	4.86

*E*_*max*_ maximum effect, *EC*_*50*_ concentration of a drug that gives half-maximal response, *E*_*0*_ concentration at baseline, *AUC*_*0–24*_ area under the concentration-time curve from 0 to 24 h, *cfu* colony forming units

## Discussion

There are several infection routes for *E. coli* when simulating colibacillosis, such as intramuscular (IM) and oral administration [16, 23]. The complicated nature of the gastrointestinal tract could result in the bacterial load in different organs after oral administration being unstable. To obtain a stable bacterial concentration in the different organs, the IM infection route was chosen. In the present study, colibacillosis was achieved through inoculation of ~ 10^7^ cfu/mL *E. coli* in chickens. However, the infection dose was lower than that used in a previous report [16], where colibacillosis was induced by oral gavage with 8 mL of *E. coli* culture containing 1.2 × 10^9^ cfu/mL. The differences in infection dose might be explained in two ways: The broilers used by Sang were 39 days old, whereas ours were 7 days old, and the inoculation method was different, oral gavage in Sang’s report vs. IM injection in this study.

The PKs of enrofloxacin have been investigated in goats, pigs, calves, horses, and sheep [24–28]. It has also been studied in chickens [29–31]. The elimination half-life (T_1/2β_) values in this study (9.78–11.4 h) were similar to those in previous reports [30]. After oral administration of 10 mg/kg of enrofloxacin, the AUC_0–24_ value (17.95 h) in this study was much lower than that reported in previously (35 h in Da Silva’s report and 25.35 in Mekala’s) [30, 32]. The difference illustrates that the pathological state affects the total amount of the drug in the blood. Although ciprofloxacin is the main active metabolite of enrofloxacin, few studies have reported the concentration of ciprofloxacin. Da Silva reported that the concentration of ciprofloxacin was lower than their limit of quantification (LOQ) (0.2 μg/mL) in healthy chickens [30]. With an LOQ of 0.02 μg/mL, the concentration of ciprofloxacin in the present study was detected even after enrofloxacin administration at dose of 1 mg/kg b.w. The biotransformation of enrofloxacin to ciprofloxacin at doses of 10 and 20 mg/kg b.w. were 7.9 and 15.3%, which was in accordance with a previous study [16]. However, the biotransformation rate was as 37% higher for the low dose (1 mg/kg b.w.). The moderate concentration of ciprofloxacin indicated that the role of ciprofloxacin should be considered in pharmacodynamic studies of enrofloxacin. A good linear relationship between dosage and AUC_0–24_ was observed for enrofloxacin and ciprofloxacin. This phenomenon was similar to that reported in previous studies [21, 33].

To the best of our knowledge, there has been no in vivo PK/PD modeling study of enrofloxacin against *E. coli* in chicks. One of the best PK/PD parameter for fluoroquinolones is AUC_0–24_/MIC [34–36], and this study further confirmed this conclusion. Both the surrogate AUC_0–24_/MIC for enrofloxacin or the combination of enrofloxacin and ciprofloxacin correlated well with effectiveness in each organ. It seems that the metabolism of ciprofloxacin has little influence on the PK/PD modeling of enrofloxacin. However, whether the active metabolite plays a role in emerging resistance or has an impact on dosing optimization needs further investigation [37], because optimization of the dosing regimen involves not only maximizing therapeutic outcome, but also minimizing the risk of developing resistance [38–40]. The values of AUC_0–24_/MIC for the bactericidal effect were 19.3–32.15 in the different organs using only the concentration of enrofloxacin for simulation, and 21.29–41.68 when using the combined concentrations of enrofloxacin and ciprofloxacin for simulation. The values were much lower than those of enrofloxacin against *E. coli* or *Salmonella typhimurium* in the intestinal content of infected chicken calculated from ex vivo PK/PD modeling (1065.93 and 719.33, respectively) [14, 16]. Several reasons may explain this significant discrepancy. First, the components of the intestinal content are very complex, a large proportion of drugs may exist in a bound form and show no antimicrobial effect; however, in our PK/PD modeling, the whole amount of the drug was involved in the AUC_0–24_/MIC calculation; therefore, the value of AUC_0–24_/MIC in the intestines may be higher than that in serum to achieve the same effect. Second, as reported previously, the discrepancy between ex vivo PK/PD modeling and in vivo PK/PD modeling was obvious [21].

Using the bacterial burden reduction in each organ as PD endpoints, the value of the PK/PD parameter, AUC_0–24_/MIC, to attain the same effect was different. The AUC_0–24_/MIC value for the bactericidal effect in the liver was higher than that in lung, and twice than that in blood. This phenomenon was also observed in our previous study. The AUC_0–24_/MIC values of danofloxacin against *Salmonella typhimurium* for the same effect in different organs also showed marked differences [22]. Similar results were also reported in other studies [20, 41]. The possible reason for these differences may lie in the differences in the initial bacterial load, the concentration diversity of drugs in each organ, and the complicated structures among different organs. The precise explanation requires further study. Usually, bacterial load reduction in a single organ is used for PD evaluation in most in vivo PK/PD studies, and for ex vivo PK/PD studies, the antibacterial effect of drugs in serum or other body fluid is used for PD calculation [12, 40, 42–47]. However, according to our results, for a systemic infection by bacteria, to describe the relationship of PK and PD exactly, bacterial loading reduction in different organs, as PD endpoints, should be compared in PK/PD modeling and the selection of a target organ for PD evaluation is critical in rational dosage recommendation.

The results obtained using this model need to be validated by clinical trials in relevant animal species. However, it is still a critical step to increase our understanding of PK/PD relationships for antimicrobials. To simulate the clinical use of enrofloxacin, enrofloxacin was administrated via gavage in this study; however, the most recent basic principles of the prudent use of antimicrobials do not support the group oral administration of fluoroquinolones in animals.

## Conclusions

In conclusion, an in vivo PK/PD model of enrofloxacin against *E. coli* in seven-day-old chicks was established using bacteria loading reduction in several organs as PD endpoints. The in vivo effectiveness of enrofloxacin against *E. coli* in different organs varied with *E*_*max*_ ranging from − 4.4 to − 5.8 Log_10_ cfu/mL. Both the surrogate AUC_0–24_/MIC of enrofloxacin or the combination AUC_0–24_/MIC of enrofloxacin and ciprofloxacin correlated well with effectiveness in each organ. The combined AUC_0–24_/MIC ratios producing bactericidal and elimination effects were 21.29 and 32.13 in blood; 41.68 and 58.52 in liver; and 27.65 and 46.22 in lung, respectively. The magnitude of PK/PD index was lowest for the same effect in blood, but highest in the liver, indicating that at the same dosage, the in vivo effectiveness of enrofloxacin against *E. coli* in different organs was not identical. This study emphasized the importance of target organ selection for PD evaluation in PK/PD modeling.

## Methods

### Organisms, chemicals, and animals

The clinical *E. coli* O78 strain, which was used in our previous study, was isolated from a broiler showing colibacillosis [23, 48]. The quality control standard *E. coli* strain ATCC 25922 was purchased from the Chinese Veterinary Culture Collection. The enrofloxacin reference standard and ciprofloxacin (98% purity) were purchased from Solarbio Life Sciences Co. Ltd. (Beijing, China). Acetonitrile (ACN) and methanol (MeOH) were purchased from TEDIA (Fairfield, CT, USA). All reagents used in this experiment were of high performance liquid chromatography (HPLC) grade. The culture medium used in this experiment was purchased from Hope Biol-Technology Co. Ltd. (Qingdao, China). One-day-old healthy broilers (*n* = 324), obtained from the Jiangsu Institute of Poultry Sciences (Yangzhou, China), were used in this study. These broilers were mixed sex (50:50), weighting from 35.2 to 45.6 g. A six-day acclimation period was set for animals before the study. The animals were maintained according to the National Standards for Laboratory Animals of China (GB 14925–2010). This study was approved by the Animal Experiments Ethics Committee at Yangzhou University (SYXK (Su) IACUC 2017–0045).

### Susceptibility testing

The MICs of enrofloxacin and ciprofloxacin against *E. coli* O78 and ATCC 25922 were determined using the micro-dilution method, according to the Clinical and Laboratory Standards Institute (CLSI) reference method [49]. The MIC of enrofloxacin in serum was also determined using the micro-dilution method according to a previous report [50]. To determine the minimum bactericidal concentration (MBC), 100 μL aliquots from the MIC determination procedure were diluted with Mueller-Hinton (MH) broth. The colony forming units (cfu) of each dilution were counted by spreading 100 μL dilutions on MH agar plates after 24 h of incubation at 37 °C. The lowest concentration of enrofloxacin that killed 99.9% of the bacteria was defined as the MBC. The mutant prevention concentration (MPC) determination was conducted according to a previous report [51]. Briefly, a series MH agar plates containing different drug concentrations (1 MIC to 64 MIC) were inoculated with more than 10^10^ cfu of *E. coli* and then incubated at 37 °C for 72 h. The MPC was determined as the lowest drug concentration that prevented bacterial growth.

### *E. coli* infection model

Preliminary experiments were conducted to confirm the inoculation amount. After a 6-day acclimation period, broilers were inoculated with 0.5 mL of *E. coli* culture containing ~ 10^7^ cfu/mL through intramuscular injection (IM) in the chest muscle. The clinical symptoms and pathological changes were observed. Then, animals were sacrificed by a lethal intravenous injection of beuthanasia (0.3 mL/kg) after anesthesia with ketamine-Xylazine. The bacterial load in blood, liver, and lung were determined by the agar plate dilution method at 24 h post inoculation.

### Pharmacokinetics

Enrofloxacin was administered orally to 280 infected broilers at doses of 1, 10, and 20 mg/kg body weight (b.w.). Ten blood samples were collected at each time point (0, 0.5, 1, 1.5, 2, 4, 6, 8, 12, and 24 h after administration). After sampling, animals were narcotized with ketamine-Xylazine and sacrificed by a lethal intravenous injection of beuthanasia (0.3 mL/kg). After incubation at room temperature, samples were centrifuged for 10 min at 3000×*g* to obtain serum. The serum was stored at − 20 °C until analysis. The concentrations of the drug in the serum were determined using HPLC with a fluorescence detector, as described previously, with some modifications [17, 52]. Briefly, 0.1 mL of serum was added to 1 mL of ACN containing 0.1% acetic acid, vortexed for 3 min, and then centrifuged at 12000×*g* for 10 min. The supernatant was transferred to a clean tube, dried under nitrogen, and re-dissolved with 0.1 mL 17% ACN. The sample was filtered through a 0.22-μm membrane before injecting into the HPLC apparatus. The recovery rate was between 80.2 and 91.3%, and the intra and inter coefficient of variation was less than 7%. The serum concentration–time data of enrofloxacin and ciprofloxacin for each animal was used to calculate the PK parameters in broilers using the WinNonlin software (version 6.1, Pharsight Corporation, Mountain View, CA, USA).

### Pharmacodynamics determination

Infected broilers (*n* = 54) were randomly divided into nine groups (*n* = 6 in each group) and treated with enrofloxacin for 3 successive days at doses ranging from 0 to 20 mg/kg b.w. (0, 1, 2, 5, 7.5, 10, 12.5, 15, and 20 mg/kg per day). At 24 h after the last dose, the broilers were humanely killed through a lethal intravenous injection of beuthanasia (0.3 mL/kg) after anesthesia with ketamine-Xylazine to collect blood, liver, and lung samples. The bacterial loading in each organ was determined via plating dilutions onto MH agar and counting the colonies after incubation at 37 °C for 24 h. The effectiveness of enrofloxacin was expressed as the bacterial reduction after treatment compared with that before treatment in each organ.

### Pharmacokinetics and Pharamcodynamics integration and modeling

The best PK/PD parameter for fluoroquinolones is AUC/MIC or C_max_/MIC; therefore, in the present study, we chose the AUC_0–24_/MIC method to model the PK data and in vitro PD data for enrofloxacin and its active metabolite ciprofloxacin. The sigmoid *E*_*max*_ model in the WinNonlin software (version 6.1; Pharsight) was used to simulate the relationship between AUC_0–24_/MIC of enrofloxacin, or the combination AUC_0–24_/MIC of enrofloxacin and ciprofloxacin and in vivo effectiveness. The equation for this model was as follows:

$$ E={E}_0+\frac{E_{\mathrm{max}}\times {C}_e^N}{EC_{50}^N+{C}_e^N} $$

In the above formula, E_0_ is the change in log_10_ cfu/mL or log_10_ cfu/g in the control sample (absence of drug). *E*_*max*_ is the difference in effect between the greatest amount of growth (as seen for the growth control, E_0_) and the greatest amount of killing. C_e_ is the tested AUC_0–24_/MIC ratio; EC_50_ is the AUC_0–24_/MIC value that reached 50% of the *E*_*max*_; and N is the Hill coefficient that describes the steepness of the AUC_0–24_/MIC-effect curve [52]. The in vivo antibacterial effects of enrofloxacin were quantified into three levels including: (1) 1 log10 cfu/mL killing (E = − 1), (2) bactericidal action (99.9% reduction, E = − 3), and (3) bacterial elimination (99.99% reduction, E = − 4).

### Data analysis

The PK data, PK/PD data, and PK/PD curve fitting were analyzed using the WinNonlin software (version 6.1; Pharsight). T-tests were conducted for other data using SPSS software (IBM, Armonk, NY, USA). *P* < 0.05 was considered statistically significant.

## Abbreviations

ACN: Acetonitrile
AUC_0–24_: Area under the concentrations-time curve from 0 to 24 h
CLSI: Clinical and Laboratory Standards Institute
CL_β_/F: Clearance divided by bioavailability
C_max_: Maximum serum concentration
F: Bioavailability
HPLC: High performance liquid chromatography
MBC: Minimum bactericidal concentration
MeOH: Methanol
MIC: Minimum inhibitory concnetration
MPC: Mutant prevention cocnetration
PK/PD: Pharmacokinetic/pharmacodynamic
T_1/2β_: Elimination half-life
T_max_: Time of maximum serum concentration
V_z_/F: Volume of distribution scaled by bioavailability
